# Implementing an eHealth tool to improve referral services for obstetric emergencies in Sierra Leone

**DOI:** 10.1093/eurpub/ckac129.418

**Published:** 2022-10-25

**Authors:** S Theuring, A Lewally, J Shepherd, R Williamson Taylor

**Affiliations:** 1 Institute of Tropical Medicine and International Health, Charité- Universitätsmedizin, Berlin, Germany; 2 National School of Midwifery, Ministry of Health and Sanitation, Freetown, Sierra Leone; 3 Princess Christian Maternity Hospital, Ministry of Health and Sanitation, Freetown, Sierra Leone

## Abstract

**Background:**

Maternal health remains a major issue of concern in Sierra Leone. In the main referral maternity institution, Princess Christian Maternity Hospital (PCMH), up to 25% of maternal deaths occur during or shortly after transit from another health facility. There is an urgent need to improve referral systems between peripheral health units (PHUs) and PCMH. Our aim was to pilot and evaluate an eHealth tool facilitating referral of obstetric emergency cases through effective teleconsultation between PHUs and PCMH.

**Methods:**

A web application was designed to capture unclear or complicated delivery cases at PHUs and request respective telemedical counselling from the referral institution PCMH. The eHealth tool was piloted at 10 PHUs in Western area urban and rural in August 2021. Necessary devices were provided and delivery staff was trained to use the app. In December 2021, we conducted focus group discussion with 3-6 delivery staff at five PHUs and at PCMH to evaluate utilization and outcomes of the tool.

**Results:**

All participants perceived the eHealth tool as an improvement of referral procedures. Response time from PCMH after a request for counselling from a PHU was mostly <30 minutes. The main perceived advantage of the tool was the systematic documentation of obstetric complications and procedures. This relieved staff from fear of wrong treatment accusations, and recorded communication with PCMH made processes and responsibilities transparent. Another important benefit was PCMH staff being already prepared to receive a specific emergency case after use of the app, thus reducing the ‘third delay’ within the referral facility. As a major obstacle to smooth referral despite the eHealth tool, a lacking ambulance system was mentioned as a critical gap.

**Conclusions:**

Exceedingly positive user experiences with this simple tool seem to make an expansion to more PHUs worthwhile. Benefits of using the app in more remote districts in Sierra Leone should be further investigated.

**Key messages:**

• Delivery staff in Sierra Leone was capable of using a web app for telemedical counselling in a useful and effective manner.

• The eHealth tool was perceived as very helpful in systematically and transparently documenting emergency delivery cases and treatment procedures.

